# A systematic review of corticosteroid treatment for noncritically ill patients with COVID-19

**DOI:** 10.1038/s41598-020-78054-2

**Published:** 2020-12-01

**Authors:** Hisayuki Shuto, Kosaku Komiya, Mari Yamasue, Sonoe Uchida, Takashi Ogura, Hiroshi Mukae, Kazuhiro Tateda, Kazufumi Hiramatsu, Jun-ichi Kadota

**Affiliations:** 1 Department of Respiratory Medicine and Infectious Diseases, Oita University Faculty of Medicine, 1-1 Idaigaoka, Hasama-machi, Yufu, Oita 879-5593 Japan; 2 Department of Respiratory Medicine, Kanagawa Cardiovascular and Respiratory Center, 6-16-1 Tomioka-higashi, Kanazawa-ku, Yokohama, Kanagawa 236-0051 Japan; 3 Department of Respiratory Medicine, Nagasaki University Graduate School of Biomedical Sciences, 1-7-1 Sakamoto, Nagasaki, 852-8501 Japan; 4 Department of Microbiology and Infectious Diseases, Japanese Association for Infectious Disease, Toho University School of Medicine, 6-11-1 Ohmori-nishi, Ohta-ku, Tokyo, 143-8541 Japan; 5 Department of Medical Safety Management, Oita University Faculty of Medicine, 1-1 Idaigaoka, Hasama-machi, Yufu, Oita 879-5593 Japan; 6 Nagasaki Harbor Medical Center, 6-39 Shinchi-machi, Nagasaki, 850-8555 Japan

**Keywords:** Diseases, Medical research

## Abstract

The World Health Organization (WHO) has published guidance recommending systemic corticosteroids for the treatment of patients with severe or critical COVID-19 and no corticosteroids for those with nonsevere COVID-19. Although their recommendations for critical cases were based on the results from seven randomized controlled trials (RCTs), those for noncritical cases were based on the results from only one RCT, the Randomized Evaluation of COVID-19 Therapy (RECOVERY) trial. In search of additional evidence of corticosteroids’ effect on COVID-19, we systematically reviewed controlled observational studies, besides RCTs, that assessed the impact of corticosteroid treatment on any type of mortality and/or other outcomes in noncritical patients. Of the 4037 titles and abstracts screened, we ultimately included the RECOVERY trial and five controlled observational studies using propensity score matching, (accessed on September 8, 2020). Two of the controlled observational studies assessed the association between corticosteroid treatment and in-hospital mortality, without finding statistical significance. Four of the controlled observational studies assessed corticosteroids’ effect on other outcomes, demonstrating that they were associated with reduced risk of intubation in patients requiring oxygen and with longer hospitalization and viral shedding in mild or moderate cases. These results support the WHO recommendations not to use corticosteroids for nonsevere COVID-19.

## Introduction

Coronavirus disease 2019 (COVID-19), which is caused by the severe acute respiratory syndrome coronavirus 2 (SARS-CoV-2), has spread worldwide, resulting in an ongoing pandemic. As of September 9, 2020, more than 27.4 million cases and more than 894,983 deaths have been reported^1^. No specific anti-SARS-CoV-2 drug has been identified to date, and given that it is believed that a cytokine storm—an excessive immune response—can be triggered by SARS-CoV-2 infection, corticosteroids have been widely administered to patients with COVID-19 since the early days of the pandemic, despite a lack of clear evidence of their safety and efficacy^2^.

On July 16, 2020, the Randomized Evaluation of COVID-19 Therapy (RECOVERY) collaboration group disclosed data on the efficacy of dexamethasone use in hospitalized patients with COVID-19 in a preliminary report^3^. This trial randomly assigned patients to receive dexamethasone (at a dose of 6 mg once daily) for 10 days or to receive usual care alone. In the dexamethasone group, the 28 day mortality was lower than that in the usual-care group among patients receiving invasive mechanical ventilation and among those receiving oxygen without invasive mechanical ventilation, but not among those who were receiving no respiratory support^3^. Given these results, most ongoing randomized control trials (RCTs) focusing on the effects of corticosteroids in patients with COVID-19 were suspended and their incomplete data were released because equipoise for withholding corticosteroids was no longer acceptable.

On September 2, the World Health Organization (WHO) published guidance for clinicians and healthcare decision makers on the use of corticosteroids in patients with COVID-19^4^. The recommendations were created according to a prospective meta-analysis of RCTs conducted by the WHO Rapid Evidence Appraisal for COVID-19 Therapies Working Group^5^ and two meta-analyses that were already published, with pooled data about the safety of systemic corticosteroids^6,7^. The guidance recommends to use systemic corticosteroids for the treatment of patients with severe and critical COVID-19 and not corticosteroids for the treatment of patients with nonsevere cases. However, although the recommendations for critical cases were based on the results from seven RCTs, those for noncritical cases were based on the results from only one RCT, the RECOVERY trial.

Because of the suspension of the ongoing RCTs after the RECOVERY trial, it would be technically difficult to obtain additional evidence regarding the impact of corticosteroid treatment on noncritically ill patients with COVID-19 from prospective interventional trials. Consequently, for additional evidence of corticosteroids for COVID-19, we systematically reviewed controlled observational studies that used propensity score matching (PSM), in addition to RCTs, which assessed the associations between corticosteroid treatment and any type of mortality and/or other clinical outcomes among noncritically ill patients with COVID-19.

## Methods

### Search strategy and selection criteria

This systematic review was conducted according to the guidelines of the Preferred Reporting Items for Systematic Reviews and Meta-Analyses statement and Meta-analysis of Observational Studies in Epidemiology guidelines^8,9^. To ensure timely publication, this review has not been registered in any database in advance. We searched for studies using the PubMed, medRxiv, EMBASE, and Cochrane Central Register of Controlled Trials (CENTRAL) databases. Combinations of the following search terms were applied: [(COVID-19) OR (SARS-CoV-2) OR (coronavirus disease 2019)] AND ((corticosteroids) OR (steroids) OR (methylprednisolone) OR (hydrocortisone) OR (prednisolone) OR (prednisone) OR (dexamethasone) OR (cortisol) OR (glucocorticoids)) (assessed on September 8, 2020; see the details in Supplementary Table S1).

We included RCTs or controlled observational studies that adjusted patients’ backgrounds between the corticosteroid treatment group and the control group using PSM, which is designed to detect associations between corticosteroid treatment and any type of mortality and/or other clinical outcomes including disease progression, such as the need for mechanical ventilation, viral shedding, or other objective variables among noncritically ill adults patients with COVID-19. “Noncritically ill” was defined as a condition not requiring mechanical ventilation, thus including “mild,” “moderate,” and “severe (noncritical)” cases, according to the guidelines published by the Chinese National Health Committee (CNHC) and the “Clinical management of COVID-19: Interim guidance,” published by the WHO^10,11^. The definitions of disease severity in both guidelines are mostly similar, but those of “moderate” and “severe” are slightly different, as shown in Table 1. In the review articles returned by the search criteria describing the relationship between corticosteroid treatment and clinical outcomes in patients with coronavirus infection, we also searched the reference lists for additional potentially eligible articles.

**Table 1:** The comparison of severity between guidelines from the World Health Organization and the Chinese National Health Committee.

Severity	WHO guideline	CNHC guideline
Mild	Symptomatic patients without evidence of viral pneumonia or hypoxia	Clinical symptoms are mild, and no sign of pneumonia on imaging
Moderate	Patients with clinical signs of pneumonia but no signs of severe pneumonia, including SpO_2_ ≥ 90% on room air	Showing fever and respiratory symptoms with radiological findings of pneumonia (SpO_2_ ≥ 94% on room air)
Severe	Patients with clinical signs of pneumonia (fever, cough, dyspnea, and fast breathing) plus one of the following: respiratory rate > 30 breaths/min or severe respiratory distress or SpO_2_ < 90% on room air	Patients meeting any of the following criteria: respiratory distress (respiratory rate > 30 breaths/min) or SpO_2_ ≤ 93% on room air or PaO_2_/FiO_2_ ≤ 300 mm Hg
Critical	Patients meet ARDS criteria, or patients meet Sepsis criteria or patients meet Sepsis shock criteria	Patients requiring mechanical ventilation or patients with shock or patients with another organ failure that requires ICU care

This table summarizes the clinical management of COVID-19 interim guidance issued by the WHO on May 27, 2020, and the Diagnosis and Treatment Protocol for Novel Coronavirus Pneumonia: Trial version 7 issued by the Chinese National Health Commission and State Administration of Traditional Chinese Medicine on March 3, 2020. CNHC: Chinese National Health Committee, WHO: World Health Organization.

Corticosteroids are typically administered to patients with more severe manifestations of COVID-19^2^; thus, studies that do not match patients’ backgrounds between the corticosteroid treatment group and the control group could cause significant biases, such as confounding and reverse causality. Therefore, such uncontrolled observational studies not using PSM were excluded from this review. Publications written in languages other than English, case reports or case series, studies published only in abstract form, review articles, letters, statements, editorials, guidance articles or guidelines, and clinical trial protocols were excluded. Studies including only critically ill patients or not classifying patients into critical or noncritical and those who focused on medications other than corticosteroid treatment were also excluded for being a mismatched sample or mismatched exposure/outcome, respectively.

The titles, abstracts, and full texts of articles were screened and further independently assessed by two respiratory infectious disease specialists (HS and MY, with 6 and 13 years of experience, respectively). Disagreements were resolved by a third reviewer (KK, with 16 years of experience as a clinical researcher). We extracted the following information from the included studies: study design, sample size, patient age, eligibility criteria such as severity according to the guidelines for COVID-19 by the CNHC (version 7)^10^ or WHO^11^, the type of corticosteroids, doses, timing, and duration of corticosteroid treatment, and the number of each outcome in patients treated with or without corticosteroids.

### Assessing the risk of bias

The risk of bias was assessed according to the scales of version 2 of the Cochrane risk-of-bias tool for randomized trials (RoB2) for the RCT or the Risk of Bias Assessment tool for Nonrandomized Studies (RoBANS) for the controlled observational studies^12^, as recommended by the Cochrane Handbook for Systematic Reviews of Interventions^13^.

The RCT was assessed for seven factors related to potential biases as follows: (1) random sequence generation, (2) allocation concealment, (3) selective reporting, (4) other sources of bias, (5) blinding (participants and personnel), (6) blinding (outcome assessment), and (7) incomplete outcome data. For the controlled observational studies, the following factors were assessed: (1) the selection of participants (e.g., source population clearly defined, study population described, and participation rate); (2) confounding variables (e.g., confounders defined, measured, and accounted for); (3) measurements of exposure (e.g., types of drug, dosage, timing, and duration); (4) blinding of outcome assessments (e.g., outcome defined and measured appropriately); (5) incomplete outcome data (e.g., loss to follow-up); and (6) selective outcome reporting (e.g., appropriate analyses and clear and sufficient presentation of data). Disagreements between the two investigators were resolved by the third reviewer.

## Results

### Database search

**Figure 1:**
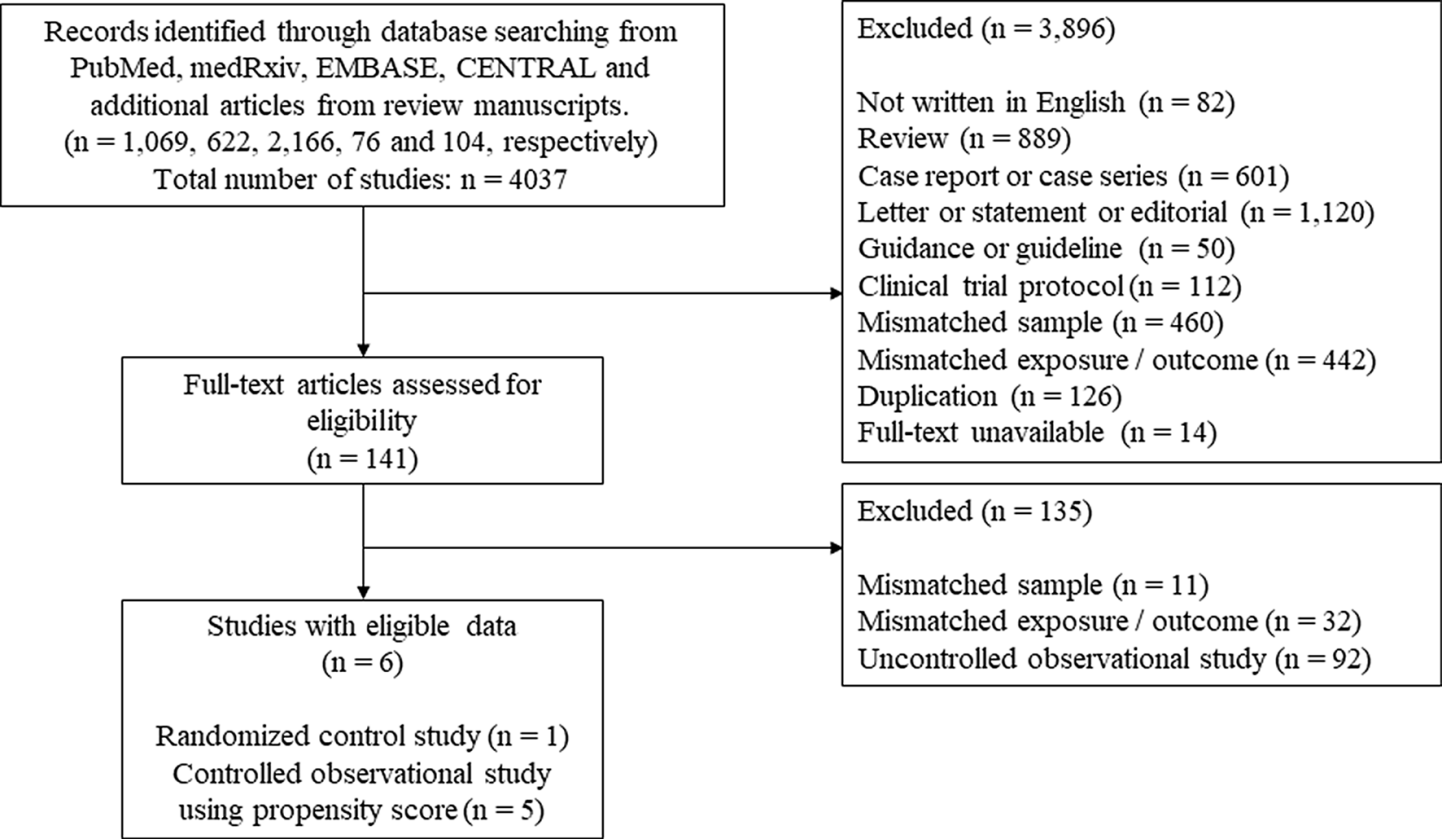
Flow diagram of study selection.

We identified 1069, 622, 2166, and 76 studies from PubMed, medRxiv, EMBASE, and CENTRAL, respectively, and 104 additional articles were identified from the review articles returned by the search criteria. We excluded 3896 studies because the abstract did not meet the inclusion criteria (Fig. 1). Of the remaining 141 studies, 135 were excluded after retrieving and inspecting the full text (see details in Supplementary Table S2). Ultimately, one RCT and five controlled observational studies using PSM were included in this systematic review. There were no overlapping cases in which more studies of the same duration were conducted in the same hospital, and no study was found that clearly stated where the patient died (e.g., ICU or general wards).

### Assessing the risk of bias

The RECOVERY trial was not double-blinded or placebo-controlled; thus, performance bias and detection bias were identified to be high on the RoB2 scale. The other assessments of bias are summarized in Supplementary Table S3. In the RoBANS scale for controlled observational studies, the average number for “high” risk of bias across the six criteria was approximately 3.8 for five controlled observational studies assessing the impact of corticosteroids on any type of mortality (Table 2). The quality of studies regarding “confounding variables” was good, whereas the quality concerning “the selection of participants”, “measurement of exposure”, and “blinding of outcome assessments” was relatively poor. The main reasons for the low quality concerning these indicators were that these were retrospective studies, and the types of drug, dose, duration, and timing of administration were heterogeneous, and there was no placebo control.

**Table 2:** Quality assessment for the controlled observational studies using the Risk of Bias Assessment tool for non-randomized controlled studies.

Author, publication or posted date	The selection of participants	Confounding variables	Measurement of exposure	Blinding of outcome assessments	Incomplete outcome data	Selective outcome reporting
Chroboczek, May 8, 2020	High	Low	High	High	Low	Low
Wu, May 11, 2020	High	Low	High	High	Low	Low
Yuan, Jun. 2, 2020	High	Low	Low	High	High	High
Ma, Aug. 12, 2020	High	Low	High	High	High	High
Li, Sep. 2, 2020	High	Low	High	High	High	Low

### The effect of corticosteroid treatment on mortality

One RCT, the RECOVERY trial (1535 patients in “no receipt of oxygen group” and 3883 in “oxygen only group”), and two controlled observational studies using PSM (475 moderate cases, and 1514 severe cases, defined by the CNHC criteria) assessed the effect of corticosteroid treatment on mortality in noncritically ill patients with COVID-19. Detailed information on the included RCT and controlled observational studies are provided in Table 3. Extensive heterogeneity regarding the type of corticosteroid, dosage, duration, and timing of corticosteroid administration was observed among these studies.

**Table 3:** Characteristics of the included studies that assessed the effect of corticosteroids on mortality.

Author, publication or posted date	Sample size (CS vs. non-CS)	Age in CS	Age in non-CS	Eligibility criteria	Types of CS	Dosage (mg/day)	Timing from symptom onset (days)	Duration (days)	Mortality type	Mortality in CS	Mortality in non-CS	*p* value
The RECOVERY Collaborative Group, Jul. 17, 2020	1535 (501 vs. 1034)	69.4 ± 17.5		“No receipt of oxygen”	DEX	6.0	6 (3–10)	10 (or until hospital discharge if sooner)	28 days	89/501 (17.8)	145/1034 (14.0)	> 0.05
	3883 (1279 vs. 2604)	66.7 ± 15.3		“Oxygen only”			9 (5–12)			298/1279 (23.3)	682/2604 (26.2)	< 0.05
Wu, May 11, 2020	1514 (531 vs. 983)	63.0 (53.0–71.0)	60.0 (50.0–69.0)	Severe (noncritical)^a^	No restriction	40.0 (37.3–57.1) MP-equivalent	n.d	6.0 (3.0–10.0)	In-hospital	83/531 (15.6)	26/983 (2.6)	0.166^b^
Li, Sep. 2, 2020	110 of 475 (55 vs. 55)	59 (46–68)	58 (43–70)	Moderate^a^	MP or PDN	MP: 20–40 PDN: 20 MP-equivalent	n.d	2 (1–5)	In-hospital	1/55 (1.8)	0/55 (0)	0.315^b^

The continuous variables are summarized as the medians (interquartile ranges) or mean (± standard deviation) and the categorical variables are presented as numbers (percentages). a: The severity setting was defined according to the guidelines published by the Chinese National Health Committee (Ver.7). b: *p* values are presented after adjustment by propensity score. CS: corticosteroid, IMV: invasive mechanical ventilation, DEX: dexamethasone, MP: methylprednisolone, PDN: prednisone, n.d.: not described.

The RECOVERY trial demonstrated that the 28 day mortality was lower in the dexamethasone group than that in the usual-care group among patients receiving oxygen without invasive mechanical ventilation (23.3% vs. 26.2%; rate ratio, 0.82; 95% CI 0.72–0.94) but not among those who were receiving no respiratory support (17.8% vs. 14.0%; rate ratio, 1.19; 95% CI 0.19–1.55)^3^.

Wu et al. retrospectively analyzed the association between corticosteroid treatment and in-hospital mortality adjusted by propensity score, dividing patients into severe (noncritical) cases and critical cases according to the CNHC guidelines^14^. Corticosteroid treatment was not significantly associated with increased in-hospital mortality in noncritically severe cases (HR 1.55; 95% CI 0.83–2.87; *p* = 0.166).

Li et al. included 475 patients with moderate COVID-19 as defined by the CNHC guidelines, and compared mortality, probable in-hospital mortality, between the corticosteroid group (n = 55) and the noncorticosteroid group (n = 55) after PSM^15^. No significant difference was observed in mortality between the groups.

### The effect of corticosteroid treatment on other clinical outcomes

One RCT, the RECOVERY trial, and four controlled observational studies using PSM (368 mild or moderate cases^16^ and 607 moderate cases^15,17^, as defined by the CNHC, and 70 patients not requiring mechanical ventilation on admission and requiring more than 3 L/min of oxygen during hospitalization to reach saturation above 93%)^18^ assessed the effect of corticosteroid treatment on other clinical outcomes in noncritically ill patients with COVID-19 as shown in Table 4.

**Table 4:** Characteristics of the included studies that assessed the effect of corticosteroids on other outcomes.

Author, publication or posted date	Sample size (CS vs. non-CS)	Age in CS	Age in non-CS	Eligibility criteria	Types of CS	Dosage (mg/day)	Timing from symptom onset (days)	Duration (days)	Outcome	Outcome in CS	Outcome in non-CS	*p* value
The RECOVERY Collaborative Group, Jul. 17, 2020	5418 (1780 vs. 3638)	n.d	n.d	“No receipt of oxygen” or “oxygen only”	DEX	6.0	N.d	10 (or until hospital discharge if sooner)	Invasive mechanical ventilation or death	456/1780 (25.6)	994/3638 (27.3)	> 0.05
									Invasive mechanical ventilation	102/1780 (5.7)	285/3638 (7.8)	< 0.05
Chroboczek May 8, 2020	70 (21 vs. 49)	61 ± 12		Cases required more than 3 l/min of oxygen at any time of hospitalization	No restriction	n.d	13 ± 4.2	n.d	Intubation	3/21 (14.3)	32/49 (65.3)	0.004^a^
Yuan, Jun. 2, 2020	70 of 132 (35 vs. 35)	48.1 (33.0–64.0)	47.7 (31.0–67.0)	Moderate^b^	MP	50.6 (40.0–50.0)	9.7 (5.8–12.0)	10.7 (8–12.3)	Time from onset of symptoms to twice continuous negative RT-PCR results	20.3 (15.2–24.8)	19.4 (11.5–28.3)	0.669^a^
									Progression to severe cases	4/35 (11.4)	1/35 (2.9)	0.356^a^
									Length of fever (days)	9.5 (6.5–12.2)	10.2 (6.8–14)	0.280^a^
									Hospital stay (days)	23.5 (19.0–29.0)	20.2 (14.0–25.3)	0.079^a^
									Secondary infection	0/35 (0)	0/35 (0)	1.000^a^
Ma, Aug. 12, 2020	120 of 368 (60 vs. 60)	47.9 ± 13.6	47.9 ± 14.0	Mild or Moderate^b^	No restriction	40^c^ MP-equivalent	N.d	6 ^c^	Length of hospitalization (days)	19.0 (14.0–25.0)	11.5 (9.0–17.0)	< 0.001^a^
	110 of 368 (55 vs. 55)	47.0 ± 13.8	47.0 ± 12.8						Length of viral shedding (days)	20.0 (16.0–25.0)	17.0 (13.0–22.0)	0.046^a^
Li, Sep. 2, 2020	110 of 475 (55 vs. 55)	59 (46–68)	58 (43–70)	Moderate^b^	MP, PDN	MP: 20–40 PDN: 20 MP-equivalent	N.d	2 (1–5)	Developed a severe disease	7/55 (12.7)	1/55 (1.8)	0.028^a^
									Duration of fever (days)	5 (4–7)	3 (1–5)	< 0.001^a^
									Virus clearance time (days)	18 (13–21)	11 (8–16)	< 0.001^a^
									Length of hospital stay (days)	23 (17–28)	15 (12–20)	< 0.001^a^

The continuous variables are summarized as the medians (interquartile ranges) or mean (± standard deviation) and the categorical variables are presented as numbers (percentages). a: *p* values are presented after adjustment by propensity score. b: The severity setting was defined according to the guidelines published by the Chinese National Health Committee (Ver.7). c: Data before propensity score matching. CS: corticosteroid, IMV: invasive mechanical ventilation, DEX: dexamethasone, ICU: intensive care unit, MP: methylprednisolone, n.d.: not described, PCR: polymerase chain reaction, PDN: prednisone.

The RECOVERY trial demonstrated that the number of patients who progressed to invasive mechanical ventilation in the dexamethasone group was significantly lower than that in the usual care group^3^. Among the controlled observational studies, Chroboczek et al. targeted patients with pneumonia not requiring mechanical ventilation on admission but requiring more than 3 L of oxygen to obtain an oxygen saturation of 92% at any time of hospitalization. This study found that corticosteroid therapy affected the risk of intubation, with a risk difference of − 47% (95% CI − 71.8 to − 22.5)^18^. Yuan et al. included moderate COVID-19 as defined by the CNHC criteria and showed no significant reduction in the number of patients who progressed to a severe condition, the length of hospitalization, the time from symptom onset to twice continuous negative reverse transcription–polymerase chain reaction, or the duration of fever^17^. Ma et al. divided COVID-19 patients into 368 “mild or moderate” cases and 82 “severe or critical” cases according to the CNHC guidelines^16^. Among “mild” or “moderate” cases, the length of hospitalization and viral shedding time of the patients in the corticosteroid group were significantly longer than that of the patients in the noncorticosteroid group after PSM. Li et al. included patients with moderate COVID-19 as defined by the CNHC criteria and found that corticosteroid treatment was associated with a larger number of patients who developed a severe disease and longer duration of fever, virus clearance time, and hospitalization^15^.

## Discussion

This systematic review included one RCT, the RECOVERY trial^3^, and five controlled observational studies using PSM^14–18^, which assessed the association between corticosteroid treatment and any type of mortality and/or other clinical outcomes among noncritically ill patients with COVID-19. Most of the results of controlled observational studies are consistent with those in the RECOVERY trial, principally contributing to the WHO recommendations issued on September 2, 2020.

The RECOVERY trial showed that corticosteroid treatment significantly reduced 28 day mortality in patients receiving oxygen without invasive mechanical ventilation but not in those who were receiving no respiratory support at randomization^3^. By contrast, Wu et al. did not find the benefit of corticosteroid treatment on in-hospital mortality in severe (noncritical) patients with COVID-19^14^. This study defined “severe” patients on the basis of the CNHC criteria, which classifies patients with a milder condition (e.g., SpO_2_ ≤ 93%) to be “severe,” in contrast to the WHO criteria (e.g., SpO_2_ ≤ 89%). Although how the RECOVERY trial determined the indication for receiving oxygen is unclear, Wu et al. might have included patients with milder manifestation, which could weaken the significance regarding the efficacy of corticosteroid treatment. However, Wu et al. demonstrated an association between corticosteroid treatment and increased in-hospital mortality even in critical cases. The authors stated “all baseline characteristics were used to construct propensity score” in the manuscript; however, there was no clear description regarding how to ensure that the calculated propensity score could appropriately predict receiving corticosteroid treatment. Technical issues in the process of propensity score creation might have led to opposite results (reverse causality), as has been observed in uncontrolled observational studies.

Li et al. showed no significant reduction in in-hospital mortality due to corticosteroid treatment among moderate patients defined by the CNHC criteria^15^, which was consistent with that in the RECOVERY trial. Notably, the mortality rates in this study (1.8% in corticosteroid group vs. 0% in noncorticosteroid group in moderate cases) were clearly lower than those in the RECOVERY trial (17.8% in the corticosteroid group vs. 14.0% in the noncorticosteroid group in “no receipt of oxygen” cases, which falls into mild or moderate cases) as shown in Table 3. Two possible reasons can be considered. First, Li et al. determined the severity using the CNHC criteria, which might have included milder patients (SpO_2_ ≥ 94%) in contrast to the criteria used in the RECOVERY trial, which might have led to lower mortality. Second, the time from symptom onset to corticosteroid administration should be discussed. In the RECOVERY trial, the median duration from symptom onset to randomization in patients not requiring oxygen at randomization was 6 days (interquartile range [IQR] 3–10), which was shorter than that in those requiring oxygen only (9 days, IQR 5–12) or invasive mechanical ventilation (13 days, IQR 8–18). Among patients who developed a severe disease, the median time from symptom onset to dyspnea ranges from 5 to 8 days and from symptom onset to acute respiratory distress syndrome 8 to 12 days^19–22^. Therefore, confirming the diagnosis of “mild or moderate” cases within a short time after symptom onset would negatively impact survival, and these patients’ clinical course should be carefully followed, and corticosteroid treatment should be at the ready once it progresses to a severe disease. Although Li et al. did not provide information regarding the time from symptom onset to admission, the included patients might have been diagnosed as “moderate” for longer after symptom onset, resulting in lower mortality than those in the RECOVERY trial.

Regarding other clinical outcomes, the results from the controlled observational study by Chroboczek et al. support the WHO recommendations to use corticosteroids for patients requiring oxygen without mechanical ventilation. Conversely, corticosteroid treatment has a negative impact on a variety of other clinical outcomes, including the number of patients who progressed to a severe disease, the length of hospitalization, and the time to viral shedding in mild or moderate cases^15,16^. In the RECOVERY trial, the results regarding secondary outcomes were not provided, dividing noncritically ill patients into the “no receipt of oxygen group” or the “oxygen only group^3^.” If the subgroup analyses are conducted in this trial, corticosteroid treatment in the “no receipt of oxygen” group might more negatively influence these outcomes than that in the “oxygen only” group.

We found some additional evidence to support the WHO recommendations that corticosteroid treatment is not recommended for mild or moderate patients with COVID-19 in terms of mortality and other clinical outcomes^15–17^. As for severe cases requiring oxygen without mechanical ventilation (severe but noncritical), no evidence was found besides the results of the RECOVERY trial^14,18^. The WHO guidance recommends corticosteroid treatment for “severe” patients as well as “critical” patients; however, patients categorized as “severe” can encompass a wide range of conditions (e.g., patients requiring anywhere from 1 to 10 L/min of oxygen). Given the evidence that corticosteroid treatment did not provide any benefit and could harm for nonsevere (mild or moderate) patients, conducting further studies subdividing the category of “severe” cases would be useful for establishing a more accurate indication for corticosteroid treatment.

This systematic review has some limitations. First, a limited number of controlled observational studies were included besides the RECOVERY trial. Several RCTs other than the RECOVERY trial, which have already been included in the WHO guidance, did not conduct a subgroup analysis. Second, significant heterogeneity was found for the type of corticosteroid, dose, duration, timing of administration, and disease severity among the included studies. The definition of “severe” condition was slightly different between the CNHC and WHO guidelines, and the “patients receiving oxygen” category used in the RECOVERY trial was not a strictly objective classification. Finally, because of the heterogeneity of the included studies, we could not conduct a meta-analysis using these collected data.

In conclusion, this systematic review focusing on corticosteroid treatment for noncritically ill patients with COVID-19 found some additional evidence to support the WHO recommendations, especially for patients not requiring any respiratory support (mild or moderate cases). However, some patients who did not require oxygen on admission might progress to a severe disease, who could then benefit from corticosteroid treatment; thus, careful monitoring is crucial for these patients. For “severe” patients (noncritical), the WHO recommendations are based on the RECOVERY trial only. The definition of the “severe” category in this trial is “receiving oxygen without mechanical ventilation,” and information regarding oxygenation status was not provided in the preliminary report. Further trials are required to determine the patients most likely to benefit from corticosteroid treatment, assessing oxygenation status more objectively, and determine the most appropriate treatment for severe (noncritical) patients with COVID-19.

## Supplementary information

Supplementary information.
