# Supplementary material for: A systematic review of corticosteroid treatment for noncritically ill patients with COVID-19
Source: Sci Rep. 2020 Dec 1;10:20935. doi: 10.1038/s41598-020-78054-2 (PMC7708623; doi:10.1038/s41598-020-78054-2)
Supplement: Supplementary file 1 — Supplementary information. [file 41598_2020_78054_MOESM1_ESM.docx]

*Supplementary files*

A systematic review of corticosteroid treatment for non-critically ill patients with COVID-19

Hisayuki Shuto MD* (1), Kosaku Komiya MD, MPH, PhD* (1), Mari Yamasue MD, PhD (1), Sonoe Uchida MD (1), Takashi Ogura MD, PhD (2), Hiroshi Mukae MD, PhD (3), Kazuhiro Tateda MD, PhD (4), Kazufumi Hiramatsu MD, PhD (1)(5), Jun-ichi Kadota MD, PhD (1)(6)

1) Department of Respiratory Medicine and Infectious Diseases, Oita University Faculty of Medicine, 1-1 Idaigaoka, Hasama-machi, Yufu, Oita 879-5593, Japan.

2) Department of Respiratory Medicine, Kanagawa Cardiovascular and Respiratory Center, 6-16-1 Tomioka-higashi, Kanazawa-ku, Yokohama, Kanagawa, 236-0051, Japan.

3) Department of Respiratory Medicine, Nagasaki University Graduate School of Biomedical Sciences, 1-7-1 Sakamoto, Nagasaki, 852-8501, Japan.

4) President of the Japanese Association for Infectious Disease, Department of Microbiology and Infectious Diseases, Toho University School of Medicine, 6-11-1 Ohmori-nishi, Ohta-ku, Tokyo, 143-8541, Japan.

5) Department of Medical Safety Management, Oita University Faculty of Medicine, 1-1 Idaigaoka, Hasama-machi, Yufu, Oita 879-5593, Japan.

6) Director, Nagasaki Harbor Medical Center, 6-39 Shinchi-machi, Nagasaki, 850-8555, Japan.

*These first authors equally contributed to this article.

Supplementary Table S1. Search strategy

| Source | Strategy |
| --- | --- |
| Pubmed, EMBASE, CENTRAL | ((COVID-19) OR (SARS-CoV-2) OR (coronavirus disease 2019)) AND ((corticosteroids) OR (steroids) OR (methylprednisolone) OR (hydrocortisone) OR (prednisolone) OR (prednisone) OR (dexamethasone) OR (cortisol) OR (glucocorticoids)) |
| MedRxiv | Due to the limited space in the search slot in MedRxiv, we modified the search strategy as follows:  ((COVID-19) OR (SARS-CoV-2) OR (coronavirus disease 2019)) AND ((corticosteroids) OR (steroids))  or  ((COVID-19) OR (SARS-CoV-2) OR (coronavirus disease 2019)) AND ((methylprednisolone) OR (hydrocortisone))  or  ((COVID-19) OR (SARS-CoV-2) OR (coronavirus disease 2019)) AND ((prednisolone) OR (prednisone) OR (dexamethasone))  or  ((COVID-19) OR (SARS-CoV-2) OR (coronavirus disease 2019)) AND ((cortisol) OR (glucocorticoids))  Then, we removed duplicates before first screening. |

There is restriction of publication date (in the Pubmed, EMBASE, CENTRAL) or posted date (in the MedRxiv): Dec/1/2019-Sep/8/2020.

Supplementary Table S2. Characteristics of excluded studies in the second screening

| Study | Database | Reason for exclusion |
| --- | --- | --- |
| Tomazini, B. M. et al. Effect of Dexamethasone on Days Alive and Ventilator-Free in Patients With Moderate or Severe Acute Respiratory Distress Syndrome and COVID-19: The CoDEX Randomized Clinical Trial. Jama, doi:10.1001/jama.2020.17021 (2020). | Pubmed | Mismatched sample |
| Dequin, P. F. et al. Effect of Hydrocortisone on 21-Day Mortality or Respiratory Support Among Critically Ill Patients With COVID-19: A Randomized Clinical Trial. Jama, doi:10.1001/jama.2020.16761 (2020). | Pubmed | Mismatched sample |
| Angus, D. C. et al. Effect of Hydrocortisone on Mortality and Organ Support in Patients With Severe COVID-19: The REMAP-CAP COVID-19 Corticosteroid Domain Randomized Clinical Trial. Jama, doi:10.1001/jama.2020.17022 (2020). | Pubmed | Mismatched sample |
| Jeronimo, C. M. P. et al. Methylprednisolone as Adjunctive Therapy for Patients Hospitalized With COVID-19 (Metcovid): A Randomised, Double-Blind, Phase IIb, Placebo-Controlled Trial. Clin Infect Dis, doi:10.1093/cid/ciaa1177 (2020). Corral, L. et al. | Pubmed | Mismatched sample |
| Nelson, B. C. et al. Clinical Outcomes Associated with Methylprednisolone in Mechanically Ventilated Patients with COVID-19. Clin Infect Dis, doi:10.1093/cid/ciaa1163 (2020). | Pubmed | Mismatched sample |
| Fernández-Cruz, A. et al. A Retrospective Controlled Cohort Study of the Impact of Glucocorticoid Treatment in SARS-CoV-2 Infection Mortality. Antimicrob Agents Chemother 64, doi:10.1128/aac.01168-20 (2020). | Pubmed | Mismatched sample |
| Lu, X., Chen, T., Wang, Y., Wang, J. & Yan, F. Adjuvant corticosteroid therapy for critically ill patients with COVID-19. Crit Care 24, 241, doi:10.1186/s13054-020-02964-w (2020). | Pubmed | Mismatched sample |
| Rodríguez-Baño, J. et al. Treatment with tocilizumab or corticosteroids for COVID-19 patients with hyperinflammatory state: a multicentre cohort study (SAM-COVID-19). Clin Microbiol Infect, doi:10.1016/j.cmi.2020.08.010 (2020). | Pubmed | Mismatched sample |
| Dubernet, A. et al. A comprehensive strategy for the early treatment of COVID-19 with azithromycin/hydroxychloroquine and/or corticosteroids: results of a retrospective observational study in the French overseas department of Reunion Island. J Glob Antimicrob Resist, doi:10.1016/j.jgar.2020.08.001 (2020). | Pubmed | Uncontrolled observational study |
| Keller, M. J. et al. Effect of systemic glucocorticoids on mortality or mechanical ventilation in patients with COVID-19. J Hosp Med 15, 489-493, doi:10.12788/jhm.3497 (2020). | Pubmed | Uncontrolled observational study |
| Liu, F. et al. Clinical characteristics and corticosteroids application of different clinical types in patients with Coronavirus disease 2019. Sci Rep 10, 13689, doi:10.1038/s41598-020-70387-2 (2020). | Pubmed | Uncontrolled observational study |
| Ma, Q. et al. Corticosteroid therapy for patients with severe novel Coronavirus disease 2019. Eur Rev Med Pharmacol Sci 24, 8194-8201, doi:10.26355/eurrev_202008_22508 (2020). | Pubmed | Uncontrolled observational study |
| Berenguer, J. et al. Characteristics and predictors of death among 4,035 consecutively hospitalized patients with COVID-19 in Spain. Clin Microbiol Infect, doi:10.1016/j.cmi.2020.07.024 (2020). | Pubmed | Uncontrolled observational study |
| Hu, F. et al. Corticosteroid, oseltamivir and delayed admission are independent risk factors for prolonged viral shedding in patients with Coronavirus disease 2019. Clin Respir J, doi:10.1111/crj.13243 (2020). | Pubmed | Uncontrolled observational study |
| Liu, J. et al. Clinical outcomes of COVID-19 in Wuhan, China: A large cohort study. Ann Intensive Care 10, 99, doi:10.1186/s13613-020-00706-3 (2020). | Pubmed | Uncontrolled observational study |
| Hu, Y. et al. Clinical efficacy of glucocorticoid on the treatment of patients with COVID-19 pneumonia: A single-center experience. Biomed Pharmacother 130, 110529, doi:10.1016/j.biopha.2020.110529 (2020). | Pubmed | Uncontrolled observational study |
| Hu, Z. et al. Clinical use of short-course and low-dose corticosteroids in patients with non-severe COVID-19 during pneumonia progression. Front Public Health 8, 355, doi:10.3389/fpubh.2020.00355 (2020). | Pubmed | Uncontrolled observational study |
| Ramiro, S. et al. Historically controlled comparison of glucocorticoids with or without tocilizumab versus supportive care only in patients with COVID-19-associated cytokine storm syndrome: results of the CHIC study. Ann Rheum Dis 79, 1143-1151, doi:10.1136/annrheumdis-2020-218479 (2020). | Pubmed | Uncontrolled observational study |
| Chopra, A. et al. Corticosteroid administration is associated with improved outcome in patients with severe acute respiratory syndrome Coronavirus 2-related acute respiratory distress syndrome. Crit Care Explor 2, e0143, doi:10.1097/cce.0000000000000143 (2020). | Pubmed | Uncontrolled observational study |
| Li, T. Z. et al. Duration of SARS-CoV-2 RNA shedding and factors associated with prolonged viral shedding in patients with COVID-19. J Med Virol, doi:10.1002/jmv.26280 (2020). | Pubmed | Uncontrolled observational study |
| Bani-Sadr, F. et al. Corticosteroid therapy for patients with COVID-19 pneumonia: A before-after study. Int J Antimicrob Agents 56, 106077, doi:10.1016/j.ijantimicag.2020.106077 (2020). | Pubmed | Uncontrolled observational study |
| Chen, X. et al. Associations of clinical characteristics and treatment regimens with the duration of viral RNA shedding in patients with COVID-19. Int J Infect Dis 98, 252-260, doi:10.1016/j.ijid.2020.06.091 (2020). | Pubmed | Uncontrolled observational study |
| Wang, K. et al. Differences of severe acute respiratory syndrome Coronavirus 2 shedding duration in sputum and nasopharyngeal swab specimens among adult inpatients with Coronavirus disease 2019. Chest, doi:10.1016/j.chest.2020.06.015 (2020). | Pubmed | Mismatched exposure / outcome |
| Giacobbe, D. R. et al. Bloodstream infections in critically ill patients with COVID-19. Eur J Clin Invest, e13319, doi:10.1111/eci.13319 (2020). | Pubmed | Uncontrolled observational study |
| Yang, Q. et al. Analysis of the clinical characteristics, drug treatments and prognoses of 136 patients with Coronavirus disease 2019. J Clin Pharm Ther 45, 609-616, doi:10.1111/jcpt.13170 (2020). | Pubmed | Mismatched exposure / outcome |
| Gong, Y. et al. Effects of methylprednisolone use on viral genomic nucleic acid negative conversion and CT imaging lesion absorption in COVID-19 patients under 50 years old. J Med Virol, doi:10.1002/jmv.26052 (2020). | Pubmed | Mismatched exposure / outcome |
| Fadel, R. et al. Early short course corticosteroids in hospitalized patients with COVID-19. Clin Infect Dis, doi:10.1093/cid/ciaa601 (2020). | Pubmed | Uncontrolled observational study |
| Lian, J. et al. Epidemiological, clinical, and virological characteristics of 465 hospitalized cases of Coronavirus disease 2019 (COVID-19) from Zhejiang province in China. Influenza Other Respir Viruses 14, 564-574, doi:10.1111/irv.12758 (2020). | Pubmed | Mismatched exposure / outcome |
| Hong, K. S. et al. Clinical features and outcomes of 98 patients hospitalized with SARS-CoV-2 infection in Daegu, South Korea: A brief descriptive study. Yonsei Med J 61, 431-437, doi:10.3349/ymj.2020.61.5.431 (2020). | Pubmed | Mismatched exposure / outcome |
| Sun, L. et al. Clinical features of patients with Coronavirus disease 2019 from a designated hospital in Beijing, China. J Med Virol, doi:10.1002/jmv.25966 (2020). | Pubmed | Uncontrolled observational study |
| Wang, Y. et al. A retrospective cohort study of methylprednisolone therapy in severe patients with COVID-19 pneumonia. Signal Transduct Target Ther 5, 57, doi:10.1038/s41392-020-0158-2 (2020). | Pubmed | Uncontrolled observational study |
| Li, X. et al. Risk factors for severity and mortality in adult COVID-19 inpatients in Wuhan. J Allergy Clin Immunol 146, 110-118, doi:10.1016/j.jaci.2020.04.006 (2020). | Pubmed | Uncontrolled observational study |
| Feng, Y. et al. COVID-19 with different severities: A multicenter study of clinical features. Am J Respir Crit Care Med 201, 1380-1388, doi:10.1164/rccm.202002-0445OC (2020). | Pubmed | Uncontrolled observational study |
| Xu, K. et al. Factors associated with prolonged viral RNA shedding in patients with Coronavirus disease 2019 (COVID-19). Clin Infect Dis 71, 799-806, doi:10.1093/cid/ciaa351 (2020). | Pubmed | Uncontrolled observational study |
| Zha, L. et al. Corticosteroid treatment of patients with Coronavirus disease 2019 (COVID-19). Med J Aust 212, 416-420, doi:10.5694/mja2.50577 (2020). | Pubmed | Uncontrolled observational study |
| Du, Y. et al. Clinical features of 85 fatal cases of COVID-19 from Wuhan. A retrospective observational study. Am J Respir Crit Care Med 201, 1372-1379, doi:10.1164/rccm.202003-0543OC (2020). | Pubmed | Mismatched exposure / outcome |
| Cao, J. et al. Clinical features and short-term outcomes of 102 patients with Coronavirus disease 2019 in Wuhan, China. Clin Infect Dis 71, 748-755, doi:10.1093/cid/ciaa243 (2020). | Pubmed | Uncontrolled observational study |
| Zheng, C. et al. Risk-adapted treatment strategy for COVID-19 patients. Int J Infect Dis 94, 74-77, doi:10.1016/j.ijid.2020.03.047 (2020). | Pubmed | Mismatched exposure / outcome |
| Jin, X. et al. Epidemiological, clinical and virological characteristics of 74 cases of Coronavirus-infected disease 2019 (COVID-19) with gastrointestinal symptoms. Gut 69, 1002-1009, doi:10.1136/gutjnl-2020-320926 (2020). | Pubmed | Mismatched exposure / outcome |
| Wan, S. et al. Clinical features and treatment of COVID-19 patients in northeast Chongqing. J Med Virol 92, 797-806, doi:10.1002/jmv.25783 (2020). | Pubmed | Mismatched exposure / outcome |
| Mo, P. et al. Clinical characteristics of refractory COVID-19 pneumonia in Wuhan, China. Clin Infect Dis, doi:10.1093/cid/ciaa270 (2020). | Pubmed | Uncontrolled observational study |
| Wu, C. et al. Risk factors associated with acute respiratory distress syndrome and death in patients with Coronavirus disease 2019 pneumonia in Wuhan, China. JAMA Intern Med 180, 1-11, doi:10.1001/jamainternmed.2020.0994 (2020). | Pubmed | Uncontrolled observational study |
| Ling, Y. et al. Persistence and clearance of viral RNA in 2019 novel Coronavirus disease rehabilitation patients. Chin Med J (Engl) 133, 1039-1043, doi:10.1097/cm9.0000000000000774 (2020). | Pubmed | Uncontrolled observational study |
| Wang, D. et al. Clinical characteristics of 138 hospitalized patients with 2019 novel Coronavirus-infected pneumonia in Wuhan, China. Jama 323, 1061-1069, doi:10.1001/jama.2020.1585 (2020). | Pubmed | Mismatched exposure / outcome |
| Lu, J. et al. Clinical characteristics and factors affecting the duration of positive nucleic acid test for patients of COVID-19 in XinYu, China. J Clin Lab Anal, e23534, doi:10.1002/jcla.23534 (2020). | Pubmed | Uncontrolled observational study |
| GLUCOCOVID: A controlled trial of methylprednisolone in adults hospitalized with COVID-19 pneumonia. medRxiv, 2020.2006.2017.20133579, doi:10.1101/2020.06.17.20133579 (2020). | MedRxiv | Mismatched sample |
| Bernaola, N. et al. Observational Study of the Efficiency of Treatments in Patients Hospitalized with Covid-19 in Madrid. medRxiv, 2020.2007.2017.20155960, doi:10.1101/2020.07.17.20155960 (2020). | MedRxiv | Mismatched sample |
| Albani, F. et al. Effect of corticosteroid treatment on 1376 hospitalized COVID-19 patients. A cohort study. medRxiv, 2020.2007.2017.20155994, doi:10.1101/2020.07.17.20155994 (2020). | MedRxiv | Mismatched sample |
| Zhao, Y. et al. An observational study of COVID-19 from a large healthcare system in northern New Jersey: Diagnosis, clinical characteristics, and outcomes. medRxiv, 2020.2008.2007.20170357, doi:10.1101/2020.08.07.20170357 (2020). | MedRxiv | Uncontrolled observational study |
| Aguas, R. et al. The potential health and economic impact of dexamethasone treatment for patients with COVID-19. medRxiv, 2020.2007.2029.20164269, doi:10.1101/2020.07.29.20164269 (2020). | MedRxiv | Mismatched exposure / outcome |
| Rahman, O. et al. Corticosteroid use in severely hypoxemic COVID-19 patients: An observational cohort analysis of dosing patterns and outcomes in the early phase of the pandemic. medRxiv, 2020.2007.2029.20164277, doi:10.1101/2020.07.29.20164277 (2020). | MedRxiv | Uncontrolled observational study |
| Ruiz-Irastorza, G. et al. Second week methyl-prednisolone pulses improve prognosis in patients with severe Coronavirus disease 2019 pneumonia: An observational comparative study using routine care data. medRxiv, 2020.2007.2016.20152868, doi:10.1101/2020.07.16.20152868 (2020). | MedRxiv | Uncontrolled observational study |
| Ooi, S. T. et al. Adjunctive corticosteroids for COVID-19: A retrospective cohort study. medRxiv, 2020.2007.2018.20157008, doi:10.1101/2020.07.18.20157008 (2020). | MedRxiv | Uncontrolled observational study |
| Monreal, E. et al. High versus standard doses of corticosteroids in COVID-19 patients with an acute respiratory distress syndrome: A controlled observational comparative study. medRxiv, 2020.2007.2017.20156315, doi:10.1101/2020.07.17.20156315 (2020). | MedRxiv | Uncontrolled observational study |
| Majmundar, M. et al. Efficacy of corticosteroids in non-intensive care unit patients with COVID-19 pneumonia from the New York Metropolitan region. medRxiv, 2020.2007.2002.20145565, doi:10.1101/2020.07.02.20145565 (2020). | MedRxiv | Uncontrolled observational study |
| Xie, Y. et al. Early diagnosis and clinical significance of acute cardiac injury - under the Iceberg: A retrospective C  cohort study of 619 non-critically ill hospitalized COVID-19 pneumonia patients. medRxiv, 2020.2007.2006.20147256, doi:10.1101/2020.07.06.20147256 (2020). | MedRxiv | Uncontrolled observational study |
| Ghalilah, K. M. et al. Effects of anticoagulants and corticosteroids therapy in patients affected by severe COVID-19 pneumonia. medRxiv, 2020.2006.2022.20134957, doi:10.1101/2020.06.22.20134957 (2020). | MedRxiv | Mismatched exposure / outcome |
| Salton, F. et al. Prolonged low-dose methylprednisolone in patients with severe COVID-19 pneumonia. medRxiv, 2020.2006.2017.20134031, doi:10.1101/2020.06.17.20134031 (2020). | MedRxiv | Uncontrolled observational study |
| Corral, L. et al. GLUCOCOVID: A controlled trial of methylprednisolone in adults hospitalized with COVID-19 pneumonia. medRxiv, 2020.2006.2017.20133579, doi:10.1101/2020.06.17.20133579 (2020). | MedRxiv | Uncontrolled observational study |
| Cao, C. et al. Clinical features and predictors for patients with severe SARS-CoV-2 pneumonia: A retrospective multicenter cohort study. medRxiv, 2020.2006.2001.20119032, doi:10.1101/2020.06.01.20119032 (2020). | MedRxiv | Uncontrolled observational study |
| Heili-Frades, S. et al. COVID-19 outcomes in 4712 consecutively confirmed SARS-CoV2 cases in the city of Madrid. medRxiv, 2020.2005.2022.20109850, doi:10.1101/2020.05.22.20109850 (2020). | MedRxiv | Uncontrolled observational study |
| Garibaldi, B. T. et al. Patient trajectories and risk factors for severe outcomes among persons hospitalized for COVID-19 in the Maryland/DC region. medRxiv, 2020.2005.2024.20111864, doi:10.1101/2020.05.24.20111864 (2020). | MedRxiv | Mismatched exposure / outcome |
| Wang, H. et al. Progression, recovery and fatality in patients with SARS-CoV-2 related pneumonia in Wuhan, China: A single-centered, retrospective, observational study. medRxiv, 2020.2005.2012.20099739, doi:10.1101/2020.05.12.20099739 (2020). | MedRxiv | Uncontrolled observational study |
| Prieto-Alhambra, D. et al. Hospitalization and 30-day fatality in 121,263 COVID-19 outpatient cases. medRxiv, 2020.2005.2004.20090050, doi:10.1101/2020.05.04.20090050 (2020). | MedRxiv | Mismatched exposure / outcome |
| Allenbach, Y. et al. Multivariable prediction model of intensive care unit transfer and death: A French prospective cohort study of COVID-19 patients. medRxiv, 2020.2005.2004.20090118, doi:10.1101/2020.05.04.20090118 (2020). | MedRxiv | Mismatched exposure / outcome |
| Wang, D. et al. No clear benefit to the use of corticosteroid as treatment in adult patients with Coronavirus disease 2019: A retrospective cohort study. medRxiv, 2020.2004.2021.20066258, doi:10.1101/2020.04.21.20066258 (2020). | MedRxiv | Uncontrolled observational study |
| Zheng, Y. et al. Clinical characteristics of 34 COVID-19 patients admitted to ICU in Hangzhou, China. medRxiv, 2020.2004.2012.20062604, doi:10.1101/2020.04.12.20062604 (2020). | MedRxiv | Mismatched exposure / outcome |
| Jiang, X. et al. Clinical features and management of severe COVID-19: A retrospective study in Wuxi, Jiangsu province, China. medRxiv, 2020.2004.2010.20060335, doi:10.1101/2020.04.10.20060335 (2020). | MedRxiv | Mismatched exposure / outcome |
| Chen, X. et al. Associations of clinical characteristics and antiviral drugs with viral RNA clearance in patients with COVID-19 in Guangzhou, China: A retrospective cohort study. medRxiv, 2020.2004.2009.20058941, doi:10.1101/2020.04.09.20058941 (2020). | MedRxiv | Uncontrolled observational study |
| Chen, M. et al. Key to successful treatment of COVID-19: Accurate identification of severe risks and early intervention of disease progression. medRxiv, 2020.2004.2006.20054890, doi:10.1101/2020.04.06.20054890 (2020). | MedRxiv | Uncontrolled observational study |
| Fu, S. et al. Virologic and clinical characteristics for prognosis of severe COVID-19: A retrospective observational study in Wuhan, China. medRxiv, 2020.2004.2003.20051763, doi:10.1101/2020.04.03.20051763 (2020). | MedRxiv | Uncontrolled observational study |
| Hu, L. et al. Risk Factors associated with clinical outcomes in 323 COVID-19 patients in Wuhan, China. medRxiv, 2020.2003.2025.20037721, doi:10.1101/2020.03.25.20037721 (2020). | MedRxiv | Uncontrolled observational study |
| Fan, L. et al. Medical treatment of 55 patients with COVID-19 from seven cities in northeast China who fully recovered: A single-center, retrospective, observational study. medRxiv, 2020.2003.2028.20045955, doi:10.1101/2020.03.28.20045955 (2020). | MedRxiv | Mismatched exposure / outcome |
| Chen, X. et al. Hypertension and diabetes delay the viral clearance in COVID-19 patients. medRxiv, 2020.2003.2022.20040774, doi:10.1101/2020.03.22.20040774 (2020). | MedRxiv | Uncontrolled observational study |
| Luo, X. et al. Characteristics of patients with COVID-19 during epidemic ongoing outbreak in Wuhan, China. medRxiv, 2020.2003.2019.20033175, doi:10.1101/2020.03.19.20033175 (2020). | MedRxiv | Uncontrolled observational study |
| Wang, Y. et al. Early, low-dose and short-term application of corticosteroid treatment in patients with severe COVID-19 pneumonia: Single-center experience from Wuhan, China. medRxiv, 2020.2003.2006.20032342, doi:10.1101/2020.03.06.20032342 (2020). | MedRxiv | Uncontrolled observational study |
| Xu, Y. et al. Clinical characteristics of SARS-CoV-2 pneumonia compared to controls in Chinese Han population. medRxiv, 2020.2003.2008.20031658, doi:10.1101/2020.03.08.20031658 (2020). | MedRxiv | Mismatched exposure / outcome |
| Zhang, G. et al. Clinical features and outcomes of 221 patients with COVID-19 in Wuhan, China. medRxiv, 2020.2003.2002.20030452, doi:10.1101/2020.03.02.20030452 (2020). | MedRxiv | Uncontrolled observational study |
| Qi, D. et al. Epidemiological and clinical features of 2019-nCoV acute respiratory disease cases in Chongqing municipality, China: A retrospective, descriptive, multiple-center study. medRxiv, 2020.2003.2001.20029397, doi:10.1101/2020.03.01.20029397 (2020). | MedRxiv | Mismatched exposure / outcome |
| Liu, Y. et al. Clinical features and progression of acute respiratory distress syndrome in Coronavirus disease 2019. medRxiv, 2020.2002.2017.20024166, doi:10.1101/2020.02.17.20024166 (2020). | MedRxiv | Uncontrolled observational study |
| Lei, L. & Jian-ya, G. Clinical characteristics of 51 patients discharged from hospital with COVID-19 in Chongqing, China. medRxiv, 2020.2002.2020.20025536, doi:10.1101/2020.02.20.20025536 (2020). | MedRxiv | Mismatched exposure / outcome |
| Liao, X. et al. Critical care for severe COVID-19: A population-based study from a province with low case-fatality rate in China. medRxiv, 2020.2003.2022.20041277, doi:10.1101/2020.03.22.20041277 (2020). | MedRxiv | Mismatched exposure / outcome |
| Giacomelli, A. et al. 30-day mortality in patients hospitalized with COVID-19 during the first wave of the Italian epidemic: A prospective cohort study. Pharmacol Res 158, 104931, doi:10.1016/j.phrs.2020.104931 (2020). | EMBASE | Mismatched exposure / outcome |
| Zhao, J., Gao, H. Y., Feng, Z. Y. & Wu, Q. J. A Retrospective analysis of the clinical and epidemiological characteristics of COVID-19 patients in Henan provincial people's hospital, Zhengzhou, China. Front Med (Lausanne) 7, 286, doi:10.3389/fmed.2020.00286 (2020). | EMBASE | Uncontrolled observational study |
| Yao, Q. et al. A retrospective study of risk factors for severe acute respiratory syndrome Coronavirus 2 infections in hospitalized adult patients. Pol Arch Intern Med 130, 390-399, doi:10.20452/pamw.15312 (2020). | EMBASE | Uncontrolled observational study |
| Chen, X. et al. Associations of clinical characteristics and treatment regimens with the duration of viral RNA shedding in patients with COVID-19. Int J Infect Dis 98, 252-260, doi:10.1016/j.ijid.2020.06.091 (2020). | EMBASE | Uncontrolled observational study |
| Huang, Q. et al. Clinical characteristics and drug therapies in patients with the common-type Coronavirus disease 2019 in Hunan, China. Int J Clin Pharm 42, 837-845, doi:10.1007/s11096-020-01031-2 (2020). | EMBASE | Mismatched exposure / outcome |
| Shi, D. et al. Clinical characteristics and factors associated with long-term viral excretion in patients with severe acute respiratory syndrome Coronavirus 2 Infection: A single-center 28-day study. J Infect Dis 222, 910-918, doi:10.1093/infdis/jiaa388 (2020). | EMBASE | Uncontrolled observational study |
| Khamis, F. et al. Clinical characteristics and outcomes of the first 63 adult patients hospitalized with COVID-19: An experience from Oman. J Infect Public Health 13, 906-913, doi:10.1016/j.jiph.2020.06.002 (2020). | EMBASE | Uncontrolled observational study |
| Huang, M. et al. Clinical characteristics and predictors of disease progression in severe patients with COVID-19 infection in Jiangsu province, China: A descriptive study. Am J Med Sci 360, 120-128, doi:10.1016/j.amjms.2020.05.038 (2020). | EMBASE | Mismatched exposure / outcome |
| Chen, Y. et al. Clinical characteristics and treatment of critically ill patients with COVID-19 in Hebei. Ann Palliat Med 9, 2118-2130, doi:10.21037/apm-20-1273 (2020) | EMBASE | Uncontrolled observational study |
| Wang, D. et al. Clinical characteristics of 138 hospitalized patients with 2019 novel Coronavirus-infected pneumonia in Wuhan, China. Jama 323, 1061-1069, doi:10.1001/jama.2020.1585 (2020). | EMBASE | Uncontrolled observational study |
| Zhang, S. Y. et al. Clinical characteristics of different subtypes and risk factors for the severity of illness in patients with COVID-19 in Zhejiang, China. Infect Dis Poverty 9, 85, doi:10.1186/s40249-020-00710-6 (2020). | EMBASE | Uncontrolled observational study |
| Huang, C. et al. Clinical features of patients infected with 2019 novel Coronavirus in Wuhan, China. Lancet 395, 497-506, doi:10.1016/s0140-6736(20)30183-5 (2020). | EMBASE | Mismatched exposure / outcome |
| Sun, L. et al. Clinical features of patients with Coronavirus disease 2019 from a designated hospital in Beijing, China. J Med Virol, doi:10.1002/jmv.25966 (2020). | EMBASE | Uncontrolled observational study |
| Hu, F. et al. Corticosteroid, oseltamivir and delayed admission are independent risk factors for prolonged viral shedding in patients with Coronavirus disease 2019. Clin Respir J, doi:10.1111/crj.13243 (2020). | EMBASE | Uncontrolled observational study |
| Cai, Q. et al. COVID-19 in a designated infectious diseases hospital outside Hubei province, China. Allergy 75, 1742-1752, doi:10.1111/all.14309 (2020). | EMBASE | Mismatched exposure / outcome |
| Wang, Z. H., Shu, C., Ran, X., Xie, C. H. & Zhang, L. Critically ill patients with Coronavirus disease 2019 in a designated ICU: Clinical features and predictors for mortality. Risk Manag Healthc Policy 13, 833-845, doi:10.2147/rmhp.S263095 (2020). | EMBASE | Uncontrolled observational study |
| Zhang, S. et al. Development and validation of a risk factor-based system to predict short-term survival in adult hospitalized patients with COVID-19: A multicenter, retrospective, cohort study. Crit Care 24, 438, doi:10.1186/s13054-020-03123-x (2020). | EMBASE | Mismatched exposure / outcome |
| Zhang, X. et al. Epidemiological, clinical characteristics of cases of SARS-CoV-2 infection with abnormal imaging findings. Int J Infect Dis 94, 81-87, doi:10.1016/j.ijid.2020.03.040 (2020). | EMBASE | Mismatched exposure / outcome |
| Qi, L. et al. Factors associated with the duration of viral shedding in adults with COVID-19 outside of Wuhan, China: A retrospective cohort study. Int J Infect Dis 96, 531-537, doi:10.1016/j.ijid.2020.05.045 (2020). | EMBASE | Uncontrolled observational study |
| Du, R. H. et al. Hospitalization and critical care of 109 decedents with COVID-19 pneumonia in Wuhan, China. Ann Am Thorac Soc 17, 839-846, doi:10.1513/AnnalsATS.202003-225OC (2020). | EMBASE | Uncontrolled observational study |
| Baraboutis, I. G., Gargalianos, P., Aggelonidou, E. & Adraktas, A. Initial real-life experience from a designated COVID-19 centre in Athens, Greece: A proposed therapeutic algorithm. SN Compr Clin Med, 1-5, doi:10.1007/s42399-020-00324-x (2020). | EMBASE | Mismatched exposure / outcome |
| Zhang, J., Yu, M., Tong, S., Liu, L. Y. & Tang, L. V. Predictive factors for disease progression in hospitalized patients with Coronavirus disease 2019 in Wuhan, China. J Clin Virol 127, 104392, doi:10.1016/j.jcv.2020.104392 (2020). | EMBASE | Uncontrolled observational study |
| Li, X. et al. Risk factors for severity and mortality in adult COVID-19 inpatients in Wuhan. J Allergy Clin Immunol 146, 110-118, doi:10.1016/j.jaci.2020.04.006 (2020). | EMBASE | Uncontrolled observational study |
| Chen, R. et al. Risk factors of fatal outcome in hospitalized subjects with Coronavirus disease 2019 from a nationwide analysis in China. Chest 158, 97-105, doi:10.1016/j.chest.2020.04.010 (2020). | EMBASE | Mismatched exposure / outcome |
| Shang, Y. et al. Scoring systems for predicting mortality for severe patients with COVID-19. EClinicalMedicine 24, 100426, doi:10.1016/j.eclinm.2020.100426 (2020). | EMBASE | Uncontrolled observational study |
| Wang, F. et al. The timeline and risk factors of clinical progression of COVID-19 in Shenzhen, China. J Transl Med 18, 270, doi:10.1186/s12967-020-02423-8 (2020). | EMBASE | Uncontrolled observational study |
| Huang, Y. et al. Treatment strategies of hospitalized patients with Coronavirus disease-19. Aging (Albany NY) 12, 11224-11237, doi:10.18632/aging.103370 (2020). | EMBASE | Uncontrolled observational study |
| Cen, Y. et al. Risk factors for disease progression in patients with mild to moderate coronavirus disease 2019-a multi-centre observational study. Clin Microbiol Infect 26, 1242-1247, doi:10.1016/j.cmi.2020.05.041 (2020). | EMBASE | Uncontrolled observational study |
| Chang, D. et al. Persistent Viral Presence Determines the Clinical Course of the Disease in COVID-19. J Allergy Clin Immunol Pract 8, 2585-2591.e2581, doi:10.1016/j.jaip.2020.06.015 (2020). | EMBASE | Uncontrolled observational study |
| Bhadade, R. et al. Appraisal of Critically Ill COVID-19 Patients at a Dedicated COVID Hospital. J Assoc Physicians India 68, 14-19 (2020). | EMBASE | Uncontrolled observational study |
| Feng, X. et al. Clinical Characteristics and Short-Term Outcomes of Severe Patients With COVID-19 in Wuhan, China. Front Med (Lausanne) 7, 491, doi:10.3389/fmed.2020.00491 (2020). | EMBASE | Uncontrolled observational study |
| Ferguson, J. et al. Characteristics and Outcomes of Coronavirus Disease Patients under Nonsurge Conditions, Northern California, USA, March-April 2020. Emerg Infect Dis 26, 1679-1685, doi:10.3201/eid2608.201776 (2020). | EMBASE | Uncontrolled observational study |
| Li, Y. et al. Corticosteroid prevents COVID-19 progression within its therapeutic window: a multicentre, proof-of-concept, observational study. Emerg Microbes Infect 9, 1869-1877, doi:10.1080/22221751.2020.1807885 (2020). | EMBASE | Uncontrolled observational study |
| Mikulska, M. et al. Tocilizumab and steroid treatment in patients with COVID-19 pneumonia. PLoS One 15, e0237831, doi:10.1371/journal.pone.0237831 (2020). | EMBASE | Mismatched exposure / outcome |
| Yang, X. et al. Clinical course and outcomes of critically ill patients with SARS-CoV-2 pneumonia in Wuhan, China: A single-centered, retrospective, observational study. Lancet Respir Med 8, 475-481, doi:10.1016/s2213-2600(20)30079-5 (2020). | Additional articles from review manuscripts | Uncontrolled observational study |
| Zhou, F. et al. Clinical course and risk factors for mortality of adult inpatients with COVID-19 in Wuhan, China: A retrospective cohort study. Lancet 395, 1054-1062, doi:10.1016/s0140-6736(20)30566-3 (2020). | Additional articles from review manuscripts | Uncontrolled observational study |
| Chen, T. et al. Clinical characteristics of 113 deceased patients with Coronavirus disease 2019: Retrospective study. Bmj 368, m1091, doi:10.1136/bmj.m1091 (2020). | Additional articles from review manuscripts | Uncontrolled observational study |
| Deng, Y. et al. Clinical characteristics of fatal and recovered cases of Coronavirus disease 2019 in Wuhan, China: A retrospective study. Chin Med J (Engl) 133, 1261-1267, doi:10.1097/cm9.0000000000000824 (2020). | Additional articles from review manuscripts | Uncontrolled observational study |
| Chen, N. et al. Epidemiological and clinical characteristics of 99 cases of 2019 novel coronavirus pneumonia in Wuhan, China: A descriptive study. Lancet 395, 507-513, doi:10.1016/s0140-6736(20)30211-7 (2020). | Additional articles from review manuscripts | Uncontrolled observational study |
| Wang, D. et al. Clinical course and outcome of 107 patients infected with the novel coronavirus, SARS-CoV-2, discharged from two hospitals in Wuhan, China. Crit Care 24, 188, doi:10.1186/s13054-020-02895-6 (2020). | Additional articles from review manuscripts | Uncontrolled observational study |
| Li, L. et al. Association of clinical and radiographic findings with the outcomes of 93 patients with COVID-19 in Wuhan, China. Theranostics 10, 6113-6121, doi:10.7150/thno.46569 (2020). | Additional articles from review manuscripts | Uncontrolled observational study |
| Xu, J. et al. Clinical course and predictors of 60-day mortality in 239 critically ill patients with COVID-19: A multicenter retrospective study from Wuhan, China. Crit Care 24, 394, doi:10.1186/s13054-020-03098-9 (2020). | Additional articles from review manuscripts | Uncontrolled observational study |
| Chen, L. et al. Risk factors for death in 1859 subjects with COVID-19. Leukemia 34, 2173-2183, doi:10.1038/s41375-020-0911-0 (2020). | Additional articles from review manuscripts | Uncontrolled observational study |
| Chen, F. et al. Clinical characteristics and risk factors for mortality among inpatients with COVID-19 in Wuhan, China. Clin Transl Med 10, doi:10.1002/ctm2.40 (2020). | Additional articles from review manuscripts | Uncontrolled observational study |
| Shi, M. et al. Analysis of clinical features and outcomes of 161 patients with severe and critical COVID-19: A multicenter descriptive study. J Clin Lab Anal, e23415, doi:10.1002/jcla.23415 (2020). | Additional articles from review manuscripts | Uncontrolled observational study |
| Ruan, Q., Yang, K., Wang, W., Jiang, L. & Song, J. Clinical predictors of mortality due to COVID-19 based on an analysis of data of 150 patients from Wuhan, China. Intensive Care Med 46, 846-848, doi:10.1007/s00134-020-05991-x (2020). | Additional articles from review manuscripts | Uncontrolled observational study |
| Wang, Y. et al. Clinical course and outcomes of 344 intensive care patients with COVID-19. Am J Respir Crit Care Med 201, 1430-1434, doi:10.1164/rccm.202003-0736LE (2020). | Additional articles from review manuscripts | Uncontrolled observational study |
| Pan, F. et al. Factors associated with death outcome in patients with severe coronavirus disease-19 (COVID-19): A case-control study. Int J Med Sci 17, 1281-1292, doi:10.7150/ijms.46614 (2020). | Additional articles from review manuscripts | Uncontrolled observational study |
| Javanian, M. et al. Clinical and laboratory findings from patients with COVID-19 pneumonia in Babol North of Iran: A retrospective cohort study. Rom J Intern Med 58, 161-167, doi:10.2478/rjim-2020-0013 (2020). | Additional articles from review manuscripts | Uncontrolled observational study |
| Dreher, M. et al. The characteristics of 50 hospitalized COVID-19 patients with and without ARDS. Dtsch Arztebl Int 117, 271-278, doi:10.3238/arztebl.2020.0271 (2020). | Additional articles from review manuscripts | Uncontrolled observational study |
| Nowak, B. et al. Clinical characteristics and short-term outcomes of patients with Coronavirus disease 2019: A retrospective single-center experience of a designated hospital in Poland. Pol Arch Intern Med 130, 407-411, doi:10.20452/pamw.15361 (2020). | Additional articles from review manuscripts | Uncontrolled observational study |
| Guan, W. J. et al. Clinical characteristics of Coronavirus disease 2019 in China. N Engl J Med 382, 1708-1720, doi:10.1056/NEJMoa2002032 (2020). | Additional articles from review manuscripts | Uncontrolled observational study |
| Liu, K. et al. Clinical characteristics of novel Coronavirus cases in tertiary hospitals in Hubei province. Chin Med J (Engl) 133, 1025-1031, doi:10.1097/cm9.0000000000000744 (2020). | Additional articles from review manuscripts | Uncontrolled observational study |

**Supplementary Table S3. Quality assessment for the randomized clinical trials using the scales of version 2 of the Cochrane risk-of-bias tool for randomized control trials.**

| Author, publication or posted date | Selection bias  Random sequence generation | Selection bias  Allocation concealment | Reporting bias  Selective reporting | Other bias  Other sources of  bias | Performance bias  Blinding (participants and personnel) | Detection bias  Blinding (outcome assessment) | Attrition bias  Incomplete outcome data |
| --- | --- | --- | --- | --- | --- | --- | --- |
| The RECOVERY Collaborative Group,  Jul. 17, 2020 | low | unclear | low | unclear | high | unclear | low |
